# On the Treatment of Wounds and Ulcers, and Hæmorrhage

**Published:** 1815-05

**Authors:** Richard Walker

**Affiliations:** of Oxford.


*For the Medical and Physical Journal.*

### *On the Treatment of Wounds and Ulcers, and Hæmorrhage:* by Mr. Richard Walker, of Oxford.

IT is my intention, as I stated at the commencement of these papers, both in physic and surgery, to confine myself to observations founded on my own experience; and in this, likewise, not to extend them to circumstances already known or established in either of these professions. It will, therefore, be found, that, although there may be a close ap-

 proximation proximation in some points, amidst such as are entirely new, yet even here there will be a sufficient shade of difference to constitute a degree of originality throughout; and, moreover, the whole may be relied on as the result of my own observation and experience.

The treatment of sores and ulcers of every description, is considered of so important and difficult a nature, as to occupy, in every practical work in surgery, a very considerable space, in consequence of the various divisions or heads under which they are placed, and the various modes of treatment to be adopted under each separate head.

Experience has convinced me, that, notwithstanding the labour which has been bestowed on this portion of surgery, the perfection to which it may be supposed to have arisen, and the best information to be obtained from books, the mode of treatment in such cases, even by the first professional persons, is frequently defective; and it occasionally becomes a matter of experiment to them, how to succeed in curing certain obstinate ulcers.

My opportunities of observation has enabled me to ascertain new and useful facts, sufficient to reduce this department of surgery to an almost mechanical accuracy or perfection in practice.

By the general introduction of *adhesive plaster*, which is of late years, in sores and ulcers, the practice is exceedingly improved, and much simplified. Cases, however, occasionally occur, in which adhesive plaster should be laid aside, and other means adopted.

My design here will be to shew where adhesive plaster is serviceable, and the best mode of applying it in such cases; and, likewise, where the use of it is to be dispensed with; and to point out, briefly, the best mode of management, as it has appeared to me, in every diversity of case which may occur. And I trust it will be found, by connecting these observations with those already given in a paper of mine, a few years since, in the Medical Journal, and which I have had occasion before to allude to, on the subject of the extraordinary efficacy of a "new modification of Carrot-poultice in certain Sores and Ulcers," that every intention will be quite or nearly fulfilled.

In all cases of fresh wounds, whether incised or lacerated, however extensive, (the blood-vessels, if any require it, having been previously secured by ligature,) adhesive plaster on cloth, or occasionally on black silk, is commonly the only application required throughout the cure, assisted by bandage. Likewise, in every case of simple ulcer, the application plication of adhesive plaster, using occasionally, when the cavity of the sore and quantity of discharge require it, lint, lightly laid on, and in sufficient quantity only to absorb the discharge.

In every case the lips of the sore are to be brought as close together as can be, at each dressing, and the plaster supported well by bandage; and in all instances, in parts which admit of it, as in the arm or leg, in order to produce the completest effect, the limb should be entirely surrounded by the adhesive plaster.

The usual method of applying adhesive plaster to sores of considerable extent, is to begin by applying a strip to the middle of the sore, proceeding, progressively, towards each extremity of the sore; in many cases, as in stumps after amputation, &c. this method is indispensable. The best method, however, in extensive wounds, wherever it can conveniently be done, is this:—having previously prepared a sufficient number of strips of adhesive plaster, begin the application of them at one extremity of the sore, (an assistant pressing the lips of the sore as nearly in contact as can be) and finish at the other extremity, each succeeding slip of plaster wrapping a little over the preceding one, applying, lastly, a binding strip, placed lengthways over the former, in the direction of the wound, extending somewhat beyond the former at each end. The next time invert the order of dressing, by beginning at the other extremity of the sore, renewing each strip successively, as the old one is removed; continuing this mode of dressing alternately: by this means the sore is prevented from opening again, and the external air is excluded. In cases of inflammation, as a refrigerant and discutient, a cataplasm of bread and goulard liquor, applied quite cold, or nearly so, according to circumstances, morning and evening, or oftener, if the heat of the part require it; using as a relaxant, or emollient, a cataplasm of bread and milk; and linseed flour and water, alone, or with suitable additions, as a suppurative cataplasm. And, as a purifyer for sores in a putrid, unclean, or unhealthy state, from any cause whatever, nothing is equal, as I have found by the most convincing experience, to the cataplasm of boiled carrots, prepared and applied according to the directions I have given in a small Treatise expressly on that subject. As a cicatrizer, when such an application becomes necessary, nothing is so efficacious as a weak solution of nitrate of silver.

The nitrate of silver possesses so specifically the power of cicatrizing sores, that I have repeatedly known it, when used with the intention of stimulating artificial openings, as

 fontanels, fontanels, &c. to discharge, to skin them over completely, even to the very bottom.

With respect to cerates, I shall only observe here, that considerable irritation and mischief is frequently occasioned, especially in hot weather, in consequence of their consistence not being sufficiently hard, whatever may be the ingredients they are composed of. Hence, I have been led to allow about one-fourth more of wax to a cerate to be used in summer than in the winter, and with very eminent advantage. I cannot forbear remarking here on the very pernicious effects of the adhesive plaster commonly sold under the name of *court-plaster*, which too commonly converts a slight scratch into a serious sore.

In all cases of wounds, sores, or ulcers, in the leg, rest in bed is essential to effect a cure perfectly and expeditiously; continued until the healing is become quite perfect and firm—supporting the part well with a roller for some time after. When such sores are accompanied with any considerable degree of inflammation, rest in bed is indispensible. Such inflammation is best removed by a goulard poultice, moist in consistence, and plentiful in quantity, applied every night and morning, or oftener, if the heat and pain in the part indicate it;—the temperature of the poultice should be such as to feel rather cool to the patient. It is the custom with some persons to add a small portion of camphorated spirit to the goulard liquor: this is extremely injurious, occasioning great pain, and increasing the inflammation.

It sometimes happens, though very rarely, if the above directions be attended to, that a poultice of bread and water is easier than one of bread and goulard liquor. When the inflammation has sufficiently subsided, adhesive plaster may be applied, finishing the cure with this, or goulard, or any other cerate, of a hardish consistence. Should there be a slight tendency to heat or inflammation in the part when the poultice has been left off, which is common towards evening, cloths, damped or soaked with goulard liquor, may be applied, over the dressing, observing to renew this as often as the heat of the part may indicate.

In order to heal a sore, particularly fresh incised wounds, with as small a cicatrix as possible, the propriety of which is too obvious to remark on, it is essential, that, at the first dressing, the lips of the wound be brought as closely into contact as possible, and confined in that state by adhesive plaster, sufficient in length, where the part will admit of it, to surround the limb entirely, in the manner before mentioned; and where the part does not admit of this, to make a sufficient purchase, to prevent the lips of the wound from receding.

receding. In the subsequent part, particular care must be taken that the sore remain not so long without dressing as that the lips of the wound recede, by the accumulation of the discharge loosening the hold of the plaster at the sore, and the lips forming a permanent adhesion underneath, at a distance from each other, which cannot afterwards be remedied. The observation last mentioned is of the utmost importance, in diminishing the scar left after the wound is healed, as well as in the expedition and firmness of healing. It will be proper, if sufficient time has elapsed for the wound to have become dry, to reduce it to the state of a bleeding fresh wound, by gentle irritation, in order to make the parts coalesce, especially in lacerated wounds.

It is essentially necessary, as I have found by long experience, that sores of every description, even trifling ones, should be dressed twice in the four and twenty hours, viz. early in the morning, and late in the evening, in order to go on well, and heal expeditiously and soundly. The exceptions to this rule are, in cases of fresh wounds; such cases as it would be either dangerous, too painful, or extremely inconvenient, to disturb so often; cases in which there is little or no discharge; and in those sores which are nearly healed. There is, in general, less occasion for frequent dressing during the use of adhesive plaster, than in the use of cerates, &c. Sores of all descriptions should be kept clean, and exposed to the air as little as possible at the time of dressing. The circumstance of dressing a sore every night and morning, is alone conducive, in a very essential degree, to the first of these objects. Care should be taken that the part be kept warm or cool, according to the season of the year and other circumstances; and, in very cold weather, that the dressing be somewhat warmed. Moreover, it should never be forgotten, that rest of the part, and an horizontal position, are most favourable, and, in many cases, indispensible, to the cure of sores of every description.

*Superficial excoriations*, discharging a thin acrimonious gleet or serum, most frequent in hot weather, are readily dried up and healed by the application of thin strata of dry lint, covered with goulard cerate of hardish consistence, and dressed often; and, if accompanied with a considerable degree of heat or inflammation, the dressings covered with cloths damped or wetted with goulard liquor occasionally, applied cold. In sores, however small, a biting corroding kind of pain coming on towards night, the sore having been dressed in the morning, is a sure sign that the sore requires to be fresh dressed; this corroding kind of pain evidently arising from the acrimonious effect of the matter discharged, and which ceases after the sore is fresh dressed.

Rest is so essential to the cure of ulcers, scorbutic sores, &c. that the attempt at curing sores of this description in working people, is frequently an almost endless business. Hence it is that persons of this description are so easily and speedily cured in hospitals, comparatively with those who attend daily a private surgery, following their ordinary avocations. A due application of adhesive plaster, with tight bandage, &c. may effect this in time; but, notwithstanding what may be said to the contrary, I am convinced, by repeated experience, that sores cured whilst the person is up, and following his usual occupation, are likewise not so firmly nor permanently healed as by rest; many such cases having returned to the Infirmary, and requiring, in order to make the healing sound and permanent, such management.

In every species of sore which will admit of it, and wherever situated, advantage should be taken of the application of adhesive plaster and bandage, in order to facilitate the healing of the sore, and likewise to obtain as small or narrow a cicatrix as possible. This latter precaution is particularly requisite in wounds of the face, and requires particular and constant attention; for, if the lips of the wound be once suffered to coalesce, in a separated or receded state, to the parts beneath, they cannot afterwards be brought into contact. This receding commonly arises from the discharge loosening the plaster; therefore, during this disposition of the sore, it should be dressed once, or even twice, every twenty-four hours, viz. at night as well as morning. Ligatures should never be used but in cases of absolute necessity, as in instances where the lip is divided or cut through, &c. and in large wounds, the lips of which cannot be otherwise brought and retained near together; and in these cases the ligature should always be supported by strips of adhesive plaster.*

A cataplasm or poultice may be considered as an application of a specific nature, with regard to efficacy in the cure of most ulcers and sores, which apparently resist all other means or modes of application, and, therefore, must occasionally be resorted to; the composition, consisting of articles agreeably to the nature or disposition of such sore or ulcer.

Rest in bed, with the application, every night and morn-

* For *goulard*, throughout this paper, may be substituted *saturnine*.

ing, ing, of a poultice of bread and milk, or in defect of fresh milk, bread and water, or of bread and goulard liquor, or the common linseed poultice, using either according as circumstances may indicate, will almost infallibly cure any ulcer or sore, not arising from a constitutional taint, as venereal, cancerous, scrophulous, &c. The temperature and renewal of the poultice should be regulated according to the following circumstances, viz. moderately warm, if the part be of an ordinary natural temperature, and cooler, or quite cold, if the part be unnaturally hot or intensely inflamed. The renewal should be regulated by the quantity of discharge, and according as the part may require excitement by regeneration of heat, or inversely an abstraction of heat, observing that the renewal be never seldomer than twice in every twenty-four hours.*

The linseed poultice is ordinarily used as a stimulating or suppurative poultice, and is, therefore, always applied warm, and occasionally renewed three, four, or more times in the twenty-four hours, adding occasionally to it other stimulating or suppurative ingredients, as onions, figs, &c.†

The propriety or necessity of dressing sores of every kind twice in the twenty-fours, (with the exceptions elsewhere pointed out,) whatever may be the nature of the application, viz. early in the morning and late in the evening, in order to the best management of them, as a general rule, originates entirely with myself, such mode being pursued by others only under extraordinary circumstances.

The advantages of it are, that the part is kept cleaner, too great an accumulation of discharge is prevented, which irritates and heats the sore; and, in certain habits, especially in hot weather, prevents a tendency to putrefaction, and likewise, as I have frequently observed, quickly removes it if commenced; and, upon the whole, diminishes the quantity of discharge, and promotes health and healing in the sore.

I have occasionally known even common blisters, from the application of vesicatories, in consequence of being dressed only once in twenty-four hours, in bad habits, par-

* But it must be observed, that ulcers or sores are not so firmly and uniformly healed by poulticing, as by dressing and bandage; therefore, at a certain period, the poultice should be discontinued, and those means adopted.

† Cataplasms and a more strongly stimulating power are occasionally required, as the *cataplasma cerevisiæ*, &c.; and as an antiseptic, the *cataplasma effervescens* is the ordinary application.

ticularly ticularly in hot weather, become vitiated or acrimonious, and show a strong disposition to putrescency or virulence, and which, doubtless, would have increased, had not the mode of dressing twice in the twenty-four hours been adopted, using precisely the same kind of dressing as before, which soon completely corrected such disposition, diminished the discharge, and quickly effected a cure.*

Whilst speaking of vesicatories, I shall observe, contrary to the ordinary opinion, viz. that blistering is always safe, and can never be productive of evil consequences (unless in cases where mortification may be apprehended); that I remember an instance of a young woman in an extremely irritable debilitated state, from an attack of fever of the typhoid kind, who evidently lost her life in consequence of the application of a large blister. The blister was directed *secundum artem*, i. e. according to the regular routine of practice, which is perfectly mechanical with some practitioners, for a pain in the side, without adverting to, or the least consideration of other contingent circumstances, which increased the irritability, debility, &c. to such a degree, that she actually sunk under it in a day or two and died.

The two circumstances which I have so strongly inculcated, viz. the specific nature of poultices in ulcers and sores, and dressing twice in the twenty-four hours, may be considered as axioms in the cure or management of sores ever to be kept in view; to which I might join, rest in bed, in certain cases, as not less essential.

The application of a roller is essentially necessary, both for keeping the lips from receding in fresh wounds, and, likewise, in all instances of sores and ulcers, in order to bring and keep the lips or edges of such sores towards contact as much as can be. It is truly surprising, as well as extremely satisfactory, to observe the difference between slack rolling and tight rolling in sores and ulcers, particularly in the leg; by a comparative trial, the latter will be found

* My residence in the infirmary afforded me opportunities of observation, and of ascertaining facts in various instances which cannot be obtained in private practice, nor by an ordinary attendance at an infirmary. In particular cases, I dressed patients myself twice every twenty-four hours, at regular periods, viz. about eight o'clock, A. M.; and at the same hour, P. M.; at other times, directing it to be so done. This should be a general rule with most sores, but which would be extremely irksome and inconvenient in private practice. And, if the avocations require the patient to be up and about, the sore should be dressed previously and well rolled, especially in ulcers and sores in the leg.

eminently eminently superior, and to facilitate the healing in an extraordinary degree.*

It may be necessary, however, occasionally, particularly in full habits, or elderly persons, to slacken the roller every afternoon, or towards evening; if the swelling of the part render it painfully tight, especially as tight rolling is much less required in an horizontal position.

N. B. Care must be taken in curing sores, particularly ulcers of long standing, that the lips be not brought together too forcibly by the application of adhesive plaster, least the part should retract or give way again, after that is left off; and, likewise, that the part be well supported and kept from retracting by wearing a roller for a sufficient time afterwards, and which, in some instances, should never be left off.

*Oxford;* RICHARD WALKER.

*April* 5, 1815.

*(To be continued.)*

### COLLECTANEA MEDICA,

CONSISTING OF

* It is, of course, always understood, that in ulcers and sores of any consequence in the leg, that the roller be applied from below the ankle up to the knee, especially if the patient be up and about; and that so necessary is due pressure by a roller under these circumstances, as a support to the part, and for preventing the descent of the humour, that the omission of it for a few hours only will be evidently apparent to an experienced surgeon.

